# Social deprivation as a risk factor for late presentation of proliferative diabetic retinopathy

**DOI:** 10.2147/OPTH.S73272

**Published:** 2015-02-17

**Authors:** Mark Lane, Priscilla A Mathewson, Hannah E Sharma, Helen Palmer, Peter Shah, Peter Nightingale, Marie D Tsaloumas, Alastair K Denniston

**Affiliations:** 1 Queen Elizabeth Hospital Birmingham, University Hospitals Birmingham NHS Foundation Trust, Birmingham, UK; 2 NIHR Biomedical Research Centre for Ophthalmology, Moorfields Eye Hospital NHS Foundation Trust and UCL Institute of Ophthalmology, London, UK; 3 Centre for Health and Social Care Improvement, School of Health and Wellbeing, University of Wolverhampton, Wolverhampton, UK; 4 Dept of Statistics, Wellcome Trust Clinical Research Facility, Birmingham, UK; 5 School of Clinical and Experimental Medicine, College of Medical and Dental Sciences, University of Birmingham, Birmingham, UK; 6 Centre for Translational Inflammation Research, College of Medical and Dental Sciences, University of Birmingham, Birmingham, UK

**Keywords:** social deprivation, index of multiple deprivation, diabetes, proliferative diabetic retinopathy

## Abstract

**Purpose:**

The aim of this study was to determine whether social deprivation is a risk factor for late presentation of patients with proliferative diabetic retinopathy and whether it affects their access to urgent laser treatment.

**Methods:**

Using a 2:1 case: control design, 102 patients referred to a UK teaching hospital as part of the UK Diabetic Retinopathy National Screening Programme were identified for the period between 1 June 2010 to 1 June 2013. Social deprivation was scored using the Index of Multiple Deprivation 2010. Additional variables considered included age, duration of disease, ethnicity, and HbA_1c_ at time of referral.

**Results:**

The cases comprised 34 patients referred with proliferative (grade R3) retinopathy with a control group of 68 patients with lower retinopathy grades; two control patients were excluded due to incomplete data. On univariate analysis, R3 retinopathy was associated with higher social deprivation (*P*<0.001, Mann–Whitney *U*-test), and with higher HbA_1c_ (11.5% vs 8.4%; *P*<0.001, Mann–Whitney *U*-test). Forward stepwise multivariable analysis showed that the association of R3 retinopathy with deprivation was significant even after adjusting for HbA_1c_ (*P*=0.016). On univariate analysis South Asian ethnicity was also identified as being a risk factor for presentation with R3 retinopathy, but this was no longer significant when HbA_1c_ was adjusted for in a forward stepwise logistic regression analysis.

**Conclusion:**

In our cohort social deprivation appears to be associated with late presentation of proliferative diabetic retinopathy. Our study supports the need to target these groups to reduce preventable blindness and to identify strategies which overcome barriers to care.

## Introduction

Diabetic retinopathy is the leading cause of blindness in the UK’s working-age population^1^ and has been estimated to cause “legal” blindness in 1,280 people per year in the UK.^2^ It has been predicted that the number of people with diabetes within England will increase from an estimated 3.2 million in 2013 to 4.2 million by 2030.^3^

Of the various retinopathy grading systems that exist, the one adopted by the National Health Service Diabetic Eye Screening Programme within England consists of three stages:^4^–^8^ R1, R2, and R3. R1 (background) retinopathy consists of microaneurysm formation, retinal hemorrhages and cotton wool spots. The additional presence of venous beading, venous reduplication and intraretinal microvascular abnormalities are indicative of R2 (pre-proliferative) retinopathy. The classification of R3 (proliferative) retinopathy is reserved for advanced disease when there are new vessels present on the disc or elsewhere, and may include pre-retinal or vitreous hemorrhages, or pre-retinal fibrosis.^9^ Maculopathy is classified separately.

In the UK all Type 1 diabetic patients over 12 years of age^10^ and all Type 2 diabetic patients from the point of diagnosis^11^ are offered annual mydriatic digital fundus photography.^12^ Patients identified with R3 retinopathy should be seen by an ophthalmologist within 2 weeks.^10^,^11^ Patients with R3 retinopathy should receive laser therapy within 2 weeks of an ophthalmologist diagnosing the condition.^8^ Treatment with pan retinal photocoagulation (PRP) in patients with R3 retinopathy reduces retinal neo-vascularization and decreases the rate of vitreous hemorrhage and tractional retinal detachment. This has been reported to reduce severe visual loss at 2 years by up to 50%.^13^

The link between social deprivation and health is well established for a number of specific conditions such as cancers of the cervix, lung^14^ and prostate,^15^ hypertension,^16^ cardiovascular disease,^17^ diabetes,^18^ and for overall morbidity and life expectancy.^19^

Within ophthalmology higher levels of social deprivation have been associated with pathology such as acute primary angle closure glaucoma,^20^ advanced presentation of primary open angle glaucoma,^21^ presentation with a lower level of visual acuity for cataract surgery,^22^ and late presentation of anisometropic amblyopia.^23^

Longer duration of disease, poorer glycemic control, and blood pressure control have all been shown to be associated with diabetic retinopathy.^24^ The aim of this study was to investigate whether social deprivation is as an independent risk factor for the development of R3 diabetic retinopathy.

## Methods

All patients referred by the Diabetic Screening Service to the University Hospitals Birmingham National Health System Foundation Trust Eye Service are identified and key data recorded by a Diabetic Eye Screening Failsafe Coordinator. These data include: date of referral, grade of retinopathy by screener, grade of retinopathy by consultant, date of hospital appointment offered, actual date of hospital appointment, numbers of cancellations or non-attendances prior to this appointment, whether referral for laser was required, date of laser offered, actual date of laser treatment, numbers of cancellations or non-attendances prior to this laser treatment. From the period 1 June 2010 to 31 May 2013 as part of a service evaluation we identified all consecutive patients referred with R3 retinopathy that had been confirmed by an ophthalmologist according to national screening criteria.^8^,^9^,^12^ A control group was identified on a 2:1 ratio comprising randomly selected “date-matched” R1–R2 (non-R3) patients who were referred to the hospital eye service during the same period.

Hospital records were used to establish the age, sex, ethnicity, type of diabetes, the date at which the patient was diagnosed with diabetes, glycemic control (HbA_1c_), and lower super-output area (LSOA; a unit of geographical area derived from the postcode) for each patient. The patient’s LSOA was linked to Index of Multiple Deprivation (IMD) 2010 reference data to estimate the level of social deprivation.

The IMD is a well validated quantifier of socio-economic status comprising measurement of deprivation in the following seven domains: income; employment; health and disability; education; crime; barriers to housing and services; and living environment. Each LSOA in the country is given an individual score for each domain. These domains are then combined to ascertain the overall level of deprivation within the area with higher scores indicating higher levels of deprivation. IMD scores were calculated for all of our patients based on their postcodes at time of presentation and 2010 reference IMD data for the West Midlands region. The use of IMD data as a measure of social deprivation is well described within the literature. IMD data have successfully been used to highlight social deprivation as an independent risk factor for a number of primary eye conditions including: severe neovascular age-related macular degeneration,^25^ acute primary angle closure glaucoma,^20^ and presentation with a lower level of visual acuity for cataract surgery.^22^

## Results

One hundred and two “new” patients were included in this study: 34 consecutive patients presenting with R3 retinopathy and 68 “date-matched” controls with non-R3 diabetic retinopathy presenting over the same time period; all patients were referred via the diabetic screening service. Two non-R3 patients were excluded from the analysis (due to incomplete data), resulting in 34 patients with R3 retinopathy and 66 control patients (Table 1).

### Patients presenting with R3 retinopathy had higher levels of social deprivation than controls

Univariate analysis of our cohort showed that presentation with R3 retinopathy requiring laser was associated with significantly higher levels of social deprivation than presentation with non-R3 retinopathy (*P*<0.001, Mann–Whitney *U*-test)*.*

### Univariate analysis of other factors

Median HbA_1c_ was higher (11.5%; 9.8%–13.3% interquartile range [IQR]) in patients presenting with R3 retinopathy than in controls (8.4%, 7.3%–9.3% IQR; *P*<0.001, Mann–Whitney *U*-test). Medians and IQRs have been used to summarize these data as a number of the datasets in this study were skewed.

**Figure 1:**
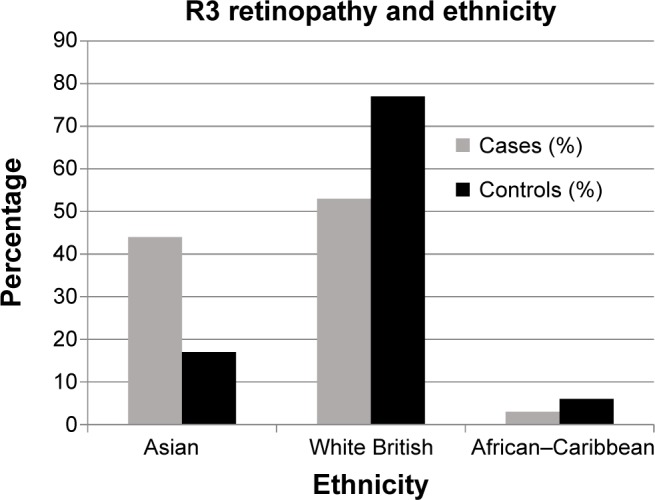
The percentage of patients in the case or control group from each ethnic category.

Ethnicity was associated with R3 retinopathy at presentation (Fisher’s exact test, *P*=0.014). South Asian ethnicity significantly increased the risk of being in the R3 group with 44% (n=15) of R3 presentations being South Asian vs 17% (n=11) in the comparator group; this compared with 53% (n=18) and 77% (n=51) respectively for White British patients; Figure 1. This meant that 58% (n=15) of Asian patients presented with R3 retinopathy compared with only 35% (n=18) of White British patients (Fisher’s exact test, *P*=0.007).

Younger age at referral was associated with R3 retinopathy at presentation. Patients with R3 retinopathy were a median of 57 years of age (48–62 IQR) at presentation and control patients were 64 years of age (49–77 IQR) (*P*=0.023, Mann–Whitney *U*-test).

Using a Fisher’s exact test there was no significant relationship between sex and R3 retinopathy (32% [n=11] in the R3 group and 47% [n=31] in the non-R3 group were female, *P*=0.201) or between type of diabetes and presence of R3 retinopathy (18% [n=6] in the R3 group and 17% [n=11] in the non-R3 group had a diagnosis of Type 1 diabetes, *P*=1.0). Similarly we did not find a significant relationship between R3 retinopathy and reported duration of diabetes (Mann–Whitney *U*-test).

### Multivariable analysis of IMD score with R3 retinopathy

Age and ethnicity were excluded from our multivariable analysis because they were not significant when HbA_1c_ was adjusted for in a forward stepwise logistic regression analysis (age; *P*=0.903, ethnicity; *P*=0.109).

Forward stepwise multivariable analysis of R3 retinopathy with IMD score and HbA_1c_ showed that the IMD score was significant after adjusting for HbA_1c_ (*P*=0.016) (Table 1).

### Comparisons made between the patients presenting with R3 retinopathy

When considering “all laser” (ie, PRP, macular or combined), there was found to be a significant difference in time to laser between the ethnicities (*P*=0.041). Pairwise comparison showed that Asian patients waited a median of 43 days (21–272 days IQR) and White British patients waited a median of only 25 days (14–39 days IQR; *P*=0.043). When looking exclusively at urgent PRP for the R3 group, the time to laser was a median (IQR) of 24 days (6–32 days) for Asian patients compared to 4 days (0–16 days) for White British patients. This difference was in part due to two patients in the Asian group who had multiple appointments arranged and yet did not attend (DNA), resulting in time to laser of 567 and 589 days, respectively. If these two “outliers” were removed, then the difference in time to surgery between Asian patients and White British patients was no longer significant (*P*=0.118).

Within the patients presenting with R3 retinopathy there was no significant association between either time to laser and IMD score (Spearman’s correlation coefficient 0.226, *P*=0.200), or frequency of DNA/cancellations and IMD score (Spearman’s correlation coefficient −0.243, *P*=0.166). There was no significant association of HbA_1c_ and time to laser within the R3 patient group. There was no correlation between IMD score and age and time to laser, or DNA/cancellation rates. There was no significant difference in total DNA or cancellation between the ethnicities.

## Discussion

This study shows that patients presenting with R3 retinopathy have higher than predicted levels of social deprivation when compared to controls. This effect is independent of HbA_1c_ level.

It has previously been demonstrated that Type 2 diabetes is more prevalent in deprived populations^26^–^28^ which may, in part, explain the increased number of deprived patients presenting with R3 retinopathy. It has also been shown that patients with higher levels of social deprivation are more likely to have worse glycemic control.^29^,^30^ This is reflected in an increase in mortality^18^,^31^ and an increase in micro vascular complications.^30^ In this study we found positive association between presence of R3 retinopathy and increased levels of HbA_1c_ (11.5% in R3 patients and 8.4% in controls). However social deprivation was found to be a risk factor for the presentation of the R3 retinopathy, even when glycemic control was adjusted for.

Previous studies have shown that an increase in social deprivation is associated with a decrease in the uptake of screening for diabetic retinopathy^32^–^34^ although it has been suggested that this inequality has reduced since the introduction of the national screening program.^35^ Scanlon et al^32^ showed that the prevalence of diabetes increased with deprivation quintile, as did the prevalence of sight threatening diabetic retinopathy, whilst the uptake of screening within this patient group was reduced. Our findings are consistent with this study with the additional finding that the effect of socio-economic status is also independent of HbA_1c_.

Our study shows that once patients had been referred from screening and attended the hospital eye service there was no significant difference in rate of DNA/cancellations or time to laser between the socio-economic groups.

### Ethnicity

The link between diabetes and ethnicity is well established. It is believed that 17% of the Asian population living in the UK has been diagnosed with Type 2 diabetes compared to 3% of the White British population.^36^ Increased levels of diabetic retinopathy in Asian patients were also documented by Raymond et al^37^ who found that Asian patients living in the Birmingham and Coventry area had an increased risk of sight threatening retinopathy when compared with White Europeans.

In this study we have shown that in our local population Asian patients have an increased risk of presenting late with R3 retinopathy with 44% (n=15) of R3 presentations in our cohort being Asian (vs only 17% [n=11] Asians in the comparator group). It should be noted that this association was no longer present after correction for HbA_1c_ levels, suggesting that this is the primary difference between these ethnic groups.

In addition to this, Asian patients waited longer for their first PRP treatment. This was in part explained by two outliers in the Asian group who had multiple DNAs resulting in delays to laser of over a year whereas no patients in the Caucasian group nor the single African–Caribbean patient had any DNA’s prior to attending their first laser treatment. This high attendance rate suggests that overall there was good engagement regarding the importance of urgent laser. Another factor appeared to be that many more laser treatments were performed on the same day as the first clinic appointment in the White British group. Since we offer this to all patients wherever possible (regardless of ethnicity, IMD status or any other demographic factor) this finding would suggest that in our community there was greater reluctance among the Asian patients to have laser performed on the same day as their first clinic appointment.

These ethnic disparities are commonly attributed to gaps in patient knowledge. Only 37% of ethnic minority patients knew that retinopathy could lead to blindness compared to 63% of the general population.^36^ This knowledge gap may be associated with language barriers. The 2001 census showed that 60% of people from ethnic minority households in the UK do not speak English as their main language at home.^36^ It is possible that the seriousness of their condition is not well conveyed through interpreters (perhaps particularly when this is a relative) resulting in non-attendance at retinopathy screening appointments.

### Age

Younger patients were more likely to present to hospital with R3 retinopathy. This may represent social factors that result in poorer engagement with the screening service or may arise from more aggressive disease in the younger age group. It has been proposed that young patients produce higher levels of vascular endothelial like growth factor^38^ and so develop R3 retinopathy at a faster rate than older patients with similar diabetic control.

### Strengths and limitations of this study

This study suggests that there is a significant association between patients presenting with active R3 retinopathy (ie, requiring urgent laser therapy) and social deprivation within a large multi-ethnic urban UK population.

In this study, we have used a case-control design to compare patients presenting to the community Diabetic Screening Service with active R3 retinopathy against “non-R3” patients being referred to the hospital eye service on a non-urgent basis during the same time period. We recognize that IMD reference data are a geographical measure of deprivation, rather than an assessment of the individual per se; however since it is uncommon for an individual’s circumstances to differ widely from the local average the IMD score is regarded as a valid tool for assessing socio-economic health care inequalities. It is important to recognize that our study only included patients who presented to the hospital eye service and as such does not include patients who either DNA screening, or who failed to attend their first hospital outpatient appointment.

It has been suggested that inequalities in health outcomes between social groups may in part be associated with increased smoking levels, poor glycemic control (in diabetic patients), raised cholesterol, obesity, lack of education, ease of access to services, a reduction in important health checks and referral bias.^39^,^40^ We recognize that whilst our multi-variate analysis accounted for glycemic control, we did not specifically look at a number of these variables. Further work is needed to determine the potential contribution of these additional factors to the development of R3 retinopathy in patients with high levels of social deprivation.

### Further work

Further work is needed to target at risk groups within our population cohort, including the socially deprived and minority ethnic groups. Minority ethnic groups need targeted support and education to help improve their glycemic control. The reluctance among the Asian patients to have laser therapy performed on the same day as their first clinic appointment needs to be formally investigated using qualitative methods since the reasons for this are unknown: possibilities range from suspicion or anxiety over the procedure itself to practical issues such as an inability to stay for the additional length of time required.

## Conclusion

This study shows that patients with a higher level of socio-economic deprivation, who are young, Asian or who have poor glycemic control are the most at risk of presenting with R3 retinopathy to screening, and thus only being referred to the hospital eye service at this advanced stage of disease. Measures that might address these issues include: targeted education on glycemic control in these high-risk groups, improved interpreter services, more flexible appointments for younger patients who work during the day, and pre-attendance telephone calls to act as reminders and to provide opportunities for discussion of the patient’s potential concerns. Before prioritizing any of these interventions we need greater clarity regarding the specific barriers that these patients encounter that result in these late presentations of R3 retinopathy, and to this end we are planning a qualitative study utilizing interviews with these late-presenting patients. It is clear that these vulnerable groups need to be prioritized by public health policy to help reduce the burden of diabetic eye disease on the individual and society.

## Figures and Tables

**Table 1:** Demographics of patients included in this study

Parameter	Case	Control
Diabetic retinopathy grading	R3	R1–R2
Number of patients	34	66
Mean (SD) age	57 years (12.3)	64 years (18.1)
Sex		
Male	23	35
Female	11	31
Ethnicity		
White British	18	51
Asian	15	11
African–Caribbean	1	4
Mean (SD) HbA_1c_	11.5% (2.29)	8.4% (1.79)

**Abbreviations:** SD, standard deviation; HbA_1c_, glycated hemoglobin.

## References

[1] Bunce C, Xing W, Wormald R (2010). Causes of blind and partial sight certifications in England and Wales: April 2007–March 2008. Eye (Lond).

[2] Scanlon PH (2008). The English national screening programme for sight-threatening diabetic retinopathy. J Med Screen.

[3] Public Health England [homepage on the Internet] (2013). Diabetes Prevalence Model for Local Authorities and CCGs.

[4] (1991). Early photocoagulation for diabetic retinopathy. ETDRS report number 9. Early Treatment Diabetic Retinopathy Study Research Group. Ophthalmology.

[5] (1991). Early Treatment Diabetic Retinopathy Study Research Group: Grading diabetic retinopathy from stereoscopic color fundus photographs: An extension of the modified Airlie House classification. Report Number 10. Ophthalmology.

[6] Bresnick GH, Mukamel DB, Dickinson JC, Cole DR (2000). A screening approach to the surveillance of patients with diabetes for the presence of vision-threatening retinopathy. Ophthalmology.

[7] Harding S, Greenwood R, Aldington S (2003). Grading and disease management in national screening for diabetic retinopathy in England and Wales. Diabet Med.

[8] The Royal College of Ophthalmologists Diabetic Retinopathy Guidelines December 2012.

[9] Taylor D (2012). Diabetic Eye Screening Revised Grading Definitions. Version 1.3.

[10] National Institute for Health and Care Excellence [homepage on the Internet] NICE Clinical Guideline 15: Type 1 Diabetes: Diagnosis and Management Of Type 1 Diabetes in Children, Young People and Adults.

[11] National Institute for Health and Care Excellence [homepage on the Internet] (2008). Royal College of Physicians National Collaborating Centre for Chronic Conditions Type 2 diabetes: national clinical guideline for management in primary and secondary care (update).

[12] Ghanchi F, Diabetic Retinopathy Guidelines Working Group (2013). The Royal College of Ophthalmologists’ clinical guidelines for diabetic retinopathy: a summary. Eye (Lond).

[13] (1981). Photocoagulation treatment of proliferative diabetic retinopathy. Clinical application of Diabetic Retinopathy Study (DRS) findings, DRS Report Number 8. The Diabetic Retinopathy Study Research Group. Ophthalmology.

[14] Shack L, Jordan C, Thomson CS, Mak V, Moller H, UK Association of Cancer Registries (2008). Variation in incidence of breast, lung and cervical cancer and malignant melanoma of skin by socioeconomic group in England. BMC Cancer.

[15] Shafique K, Morrison DS (2013). Socio-Economic Inequalities in Survival of Patients with Prostate Cancer: Role of Age and Gleason Grade at Diagnosis. PLoS One.

[16] Colhoun HM, Hemingway H, Poulter NR (1998). Socio-economic status and blood pressure: an overview analysis. J Hum Hypertens.

[17] Kaplan GA, Keil JE (1993). Socioeconomic factors and cardiovascular disease: a review of the literature. Circulation.

[18] Robinson N, LLoyd CE, Stevens LK (1998). Social deprivation and mortality in adults with diabetes mellitus. Diabet Med.

[19] Adler NE, Ostrove JM (1999). Socioeconomic Status and Health: What We Know and What We Don’t. Ann N Y Acad Sci.

[20] Nessim M, Denniston AK, Nolan W, Holder R, Shah P (2010). Research into Glaucoma And Ethnicity (ReGAE) 8: is there a relationship between social deprivation and acute primary angle closure?. Br J Ophthalmol.

[21] Fraser S, Bunce C, Wormald R, Brunner E (2001). Deprivation and late presentation of glaucoma: case-control study. BMJ.

[22] Chua PY, Mustafa MS, Scott NW, Kumarasamy M, Azuara-Blanco A (2013). Relationship between socioeconomic deprivation or urban/rural residence and visual acuity before cataract surgery in Northern Scotland. Eur J Ophthalmol.

[23] Smith LK, Thompson JR, Woodruff G, Hiscox F (1994). Social deprivation and age at presentation in amblyopia. J Public Health Med.

[24] Ya u J, Rogers SL, Kawasaki R (2012). Global prevalence and major risk factors of diabetic retinopathy. Diabetes Care.

[25] Sharma HE, Mathewson PA, Lane M (2014). The role of social deprivation in severe neovascular age-related macular degeneration. Br J Ophthalmol.

[26] Evans JM, Newton RW, Ruta DA, MacDonald TM, Morris AD (2000). Socio-economic status, obesity and prevalence of Type 1 and Type 2 diabetes mellitus. Diabet Med.

[27] Beeching NJ, Gill GV (2000). Deprivation and Type 2 diabetes mellitus prevalence. Diabet Med.

[28] Robbins JM, Vaccarino V, Zhang H, Kasl SV (2005). Socioeconomic status and diagnosed diabetes incidence. Diabetes Res Clin Pract.

[29] Weng C, Coppini DV, Sonksen PH (2000). Geographic and social factors are related to increased morbidity and mortality rates in diabetic patients. Diabet Med.

[30] Bihan H, Laurent S, Sass C (2005). Association Among Individual Deprivation, Glycemic Control, and Diabetes Complications: The EPICES score. Diabetes Care.

[31] Roper NA, Bilous RW, Kelly WF, Unwin NC, Connolly VM (2001). Excess mortality in a population with diabetes and the impact of material deprivation: longitudinal, population based study. BMJ.

[32] Scanlon PH, Carter SC, Foy C, Husband RF, Abbas J, Bachmann MO (2008). Diabetic retinopathy and socioeconomic deprivation in Gloucestershire. J Med Screen.

[33] Millett C, Dodhia H (2006). Diabetes retinopathy screening: audit of equity in participation and selected outcomes in South East London. J Med Screen.

[34] Leese GP, Boyle P, Feng Z, Emslie-Smith A, Ellis JD (2008). Screening Uptake in a Well-Established Diabetic Retinopathy Screening Program: The role of geographical access and deprivation. Diabetes Care.

[35] Gulliford MC, Dodhia H, Chamley M (2010). Socio-economic and ethnic inequalities in diabetes retinal screening. Diabet Med.

[36] diabetes.org.uk [homepage on the Internet] (2006). Diabetes and the disadvantaged: reducing health inequalities in the UK. A report by the All Parliamentary Group for Diabetes and Diabetes UK.

[37] Raymond NT, Varadhan L, Reynold DR (2009). Higher Prevalence of Retinopathy in Diabetic Patients of South Asian Ethnicity Compared With White Europeans in the Community. Diabetes Care.

[38] Rivard A, Berthou-Soulie L, Principe N (2000). Age-dependent defect in vascular endothelial growth factor expression is associated with reduced hypoxia-inducible factor 1 activity. J Biol Chem.

[39] Hippisley-Cox J, O’Hanlon S, Coupland C (2004). Association of deprivation, ethnicity, and sex with quality indicators for diabetes: population based survey of 53,000 patients in primary care. BMJ.

[40] Health and Social Care Information Centre [homepage on the Internet] (2006). Health Survey for England 2004: Volume 1. The health of minority ethnic groups.

